# Enhancing cervical cancer detection and robust classification through a fusion of deep learning models

**DOI:** 10.1038/s41598-024-61063-w

**Published:** 2024-05-11

**Authors:** Sandeep Kumar Mathivanan, Divya Francis, Saravanan Srinivasan, Vaibhav Khatavkar, Karthikeyan P, Mohd Asif Shah

**Affiliations:** 1 School of Computer Science and Engineering, Galgotias University, Greater Noida, 203201 India; 2 Department of Electronics and Communication Engineering, PSNA College of Engineering and Technology, Dindigul, India; 3 Department of Computer Science and Engineering, Vel Tech Rangarajan Dr. Sagunthala R&D Institute of Science and Technology, Chennai, India; 4 School of Computing Science and Engineering, VIT Bhopal University, Bhopal–Indore Highway Kothrikalan, Sehore, Madhya Pradesh India; 5 Department of Computer Applications, School of Computer Science Engineering and Information Systems, Vellore Institute of Technology, Vellore, Tamil Nadu 632014 India; 6 Kebri Dehar University, Kebri Dehar, Somali 250, Ethiopia; 7 Division of Research and Development, Lovely Professional University, Phagwara, Punjab 144001, India

**Keywords:** Cervical cancer, Classification, Pap smear, Deep neural network, Machine learning, Cancer, Health care, Medical research

## Abstract

Cervical cancer, the second most prevalent cancer affecting women, arises from abnormal cell growth in the cervix, a crucial anatomical structure within the uterus. The significance of early detection cannot be overstated, prompting the use of various screening methods such as Pap smears, colposcopy, and Human Papillomavirus (HPV) testing to identify potential risks and initiate timely intervention. These screening procedures encompass visual inspections, Pap smears, colposcopies, biopsies, and HPV-DNA testing, each demanding the specialized knowledge and skills of experienced physicians and pathologists due to the inherently subjective nature of cancer diagnosis. In response to the imperative for efficient and intelligent screening, this article introduces a groundbreaking methodology that leverages pre-trained deep neural network models, including Alexnet, Resnet-101, Resnet-152, and InceptionV3, for feature extraction. The fine-tuning of these models is accompanied by the integration of diverse machine learning algorithms, with ResNet152 showcasing exceptional performance, achieving an impressive accuracy rate of 98.08%. It is noteworthy that the SIPaKMeD dataset, publicly accessible and utilized in this study, contributes to the transparency and reproducibility of our findings. The proposed hybrid methodology combines aspects of DL and ML for cervical cancer classification. Most intricate and complicated features from images can be extracted through DL. Further various ML algorithms can be implemented on extracted features. This innovative approach not only holds promise for significantly improving cervical cancer detection but also underscores the transformative potential of intelligent automation within the realm of medical diagnostics, paving the way for more accurate and timely interventions.

## Introduction

Cervical cancer, a prevalent malignancy significantly affecting women, presents significant health challenges, particularly in underdeveloped nations with high morbidity and mortality rates^1^. In countries like India, cervical cancer constitutes approximately 6–29% of all female cancer diagnoses, primarily focusing on squamous cells. The disease classifies into three stages: CIN1, CIN2, and CIN3, representing mild, moderate, and severe stages, respectively^2^. Initiated by Human Papillomavirus (HPV), specifically high-risk strains, cervical cancer involves aberrant transformations in cervix cells, leading to the synthesis of E6 and E7 proteins^3^. These proteins, influencing tumor suppressor genes, play a pivotal role in cancer initiation. In the subjective field of cancer diagnosis, heavily reliant on pathologists and gynaecologists, artificial intelligence, particularly deep learning (DL), has streamlined the diagnostic process^4^. DL automates intricate feature extraction, excelling at recognizing inherent traits within images and enhancing performance^3^. As a favoured approach for cancer categorization, DL methodologies revolutionize image processing by eliminating the need for explicit feature extraction^5^. In cervical cancer diagnosis, tests like HPV testing, PAP testing, colposcopy, and biopsy are crucial, with AI increasingly playing a prominent role in prognosis and diagnostics^6^. DL’s ability to automatically classify images by learning high-level characteristics empowers pathologists in the challenging task of cancer diagnosis. Prevention and screening programs, encompassing various tests, are pivotal components in the ongoing fight against cervical cancer^7^.

While PAP smear image screening remains a primary method for addressing cervical cancer, it presents challenges. It demands a higher volume of microscopic examinations for both cancer and noncancer cases, is time-intensive, and necessitates the expertise of trained professionals^8^. Additionally, there exists a risk of overlooking positive cases when employing conventional screening techniques. Both PAP smears and HPV tests, despite their expense, offer limited sensitivity in cancer detection. Colposcopy screening serves as a vital complement to address the limitations of PAP smear images and HPV tests^9^. Early detection of cervical and other cancers becomes more feasible, even in the absence of discernible signs and symptoms. Successful screening programs hold the potential to prevent cervical cancer fatalities, ultimately reducing disease burden and suffering. Colposcopy, a widely employed surgical technique, plays a crucial role in cervical cancer screening^10^. Swift identification and categorization of this cancer type can significantly enhance the patient's overall clinical management. Numerous research publications have explored diverse methodologies within digital colposcopy to extract valuable insights from images^11^. The overarching aim of these studies is to equip healthcare practitioners with valuable resources during colposcopy examinations, catering to their varying levels of expertise. Previous research in diagnosis has harnessed computer-aided systems for a myriad of tasks, encompassing image quality enhancement and assessment, regional segmentation, image recognition, identification of unstable regions and patterns, classification of transition zone types (TZ), and cancer risk assessment. Computer-aided design (CAD) tools play a pivotal role in enhancing the quality of cervical colposcopy images, segmenting regions of concern, and pinpointing specific anomalies^12^. These strategies prove invaluable to physicians in their diagnostic processes, although they require a solid foundation of experience and skill to establish precise diagnoses. Detecting diseased areas, such as potential neoplasms, during a colposcopy examination assumes critical importance. Noteworthy examples of these abnormal regions include acetowhite areas, aberrant vascularization, mosaic patterns, and punctate lesions^13^.

**Figure 1:**
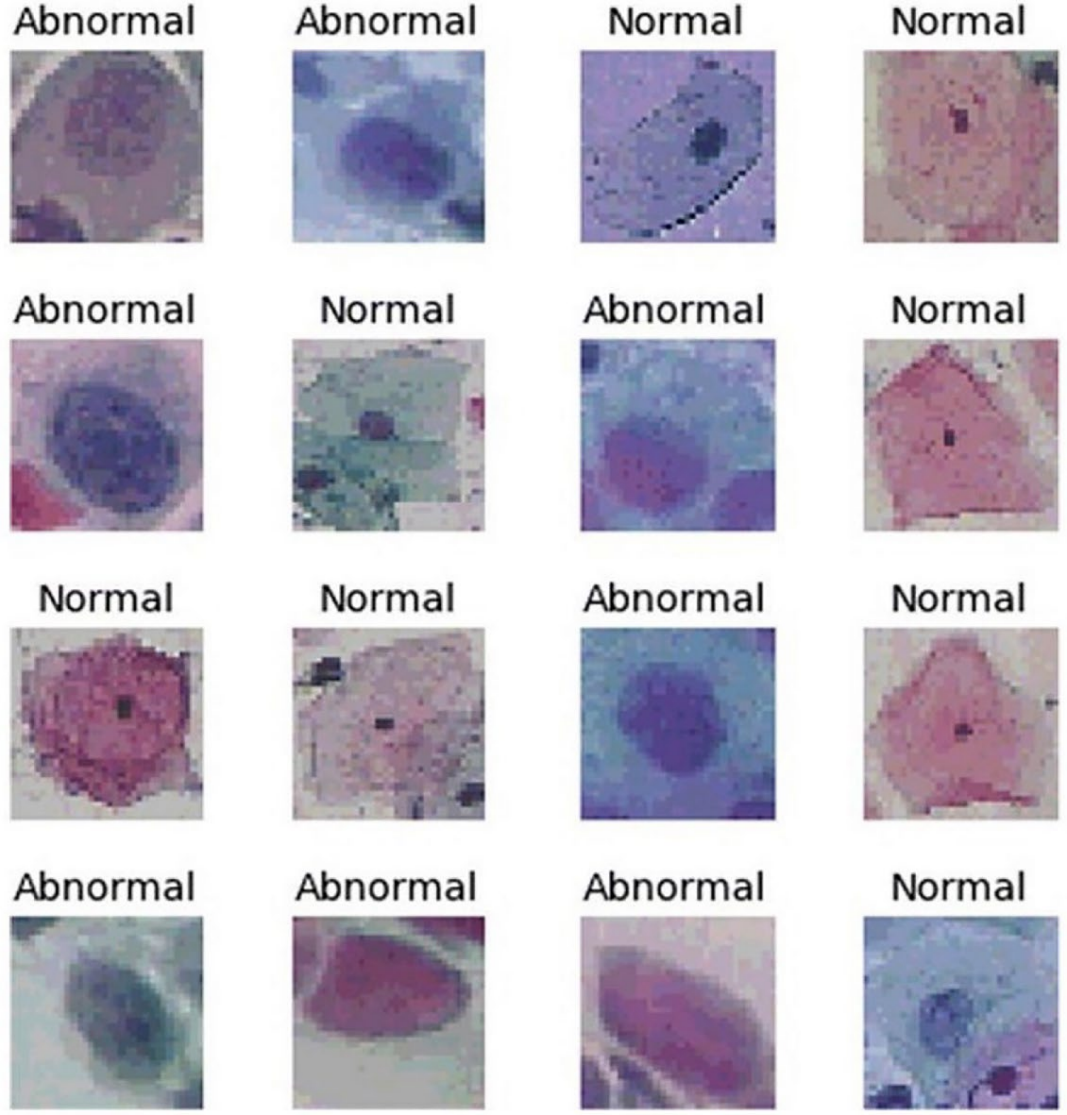
Sample normal and abnormal images from dataset.

Our proposed approach skilfully combines the power of DL with traditional machine-learning (ML) methods. We extract features by carefully collecting activation values from deep neural networks. Then, we use a different type of classifiers, such as Simple Logistic, Principal Component Analysis, and Random Tree techniques, to make accurate classifications. In our work with the SIPaKMeD dataset, we take a comprehensive approach, as shown in Fig. 1, to provide a clear overview of our system. We use DL techniques for feature extraction and employ a wide range of ML methods for classification. We thoroughly tested our system on the SIPaKMeD dataset, using a range of pre-trained models like Alexnet, Resnet-101, Resnet-152, and InceptionV3 for feature extraction. Our innovative approach consistently outperforms other models, achieving higher accuracy with the Simple Logistic model on the testing set. In this article, we introduce a hybrid methodology that combines DL and ML elements, presenting a comprehensive comparison of different pre-trained DL models. This research sheds light on their effectiveness in addressing cervical cancer classification challenges.

The paper is structured as follows: section “Introduction” provides a concise overview of key concepts. Section “Related work” delves into existing research on ML and DL methods in cervical cancer. Section “Materials and methods” offers a detailed explanation of our novel approach. Section “Experimental results and discussion” outlines the datasets used. Section “Conclusion” discusses the outcomes of our experiments. Conclusion provides a summary of the paper’s key findings and insights.

## Related work

The author introduced two deep learning convolutional neural network (CNN) architectures, namely the VGG19 (TL) model and CYENET, for the purpose of cervical cancer detection using colposcopy images. The VGG19 model is utilized as a transfer learning component within our CNN architecture. Our efforts in automatic cervical cancer classification through colposcopy images have culminated in the creation of a novel model known as the Colposcopy Ensemble Network, or CYENET. We have evaluated the predictive performance of this model, estimating its accuracy, specificity, and sensitivity. Specifically, the VGG19 (TL) model achieves a commendable 73.3% success rate in classifying the data, yielding satisfactory results overall. Furthermore, the VGG19 model, in the transfer learning context, exhibits a moderate kappa score for classification tasks. Our experimental findings underscore the excellence of the proposed CYENET model, which demonstrates remarkable sensitivity, specificity, and high kappa values, with percentages of 92.4%, 96.2%, and 88%, respectively. These results highlight the efficacy of our approach in cervical cancer detection using colposcopy images^14^. The author conducted a cervical cell categorization study using the publicly available SIPaKMeD dataset, which contains five cell classifications: superficial-intermediate, parabasal, koilocytotic, metaplastic, and dyskeratotic. The study employed a CNN to distinguish between healthy cervical cells, cells with precancerous abnormalities, and benign cells. Following the segmentation of Pap smear images, cervical cells in the resulting enhanced images were analyzed using a deep CNN with four convolutional layers and obtained an accuracy rate of 91.13%^15^.

The aim of author research was to leverage deep learning techniques to establish an integrated framework for automated cervix type classification and cervical cancer detection. To achieve this, we collected a comprehensive dataset consisting of 4005 colposcopy photos and 915 histopathology images from diverse clinical sources and publicly available databases. To enhance our approach, we developed a streamlined MobileNetv2-YOLOv3 model, which was trained and validated to identify the transformation region within cervix images. This region served as the Region of Interest (ROI) for subsequent classification. The ROI extraction process exhibited exceptional performance, with a mean average accuracy (mAP) of 99.88%. Furthermore, cervix type and cervical cancer classification tests achieved high accuracy rates, with scores of 96.84% and 94.50%, respectively. These results demonstrate the effectiveness of the approach in automating cervix type categorization and cervical cancer detection^16^. When LBCs and biopsies were initially tested using a modified general primer for HPV PCR, any samples that were found to be HPV-negative underwent subsequent whole genome sequencing. Out of the 1052 pre-CIN3+ LBC samples, HPV was detected in an impressive 97.0% (1020 samples) using the Cobas 4800 assay. Additionally, nine samples revealed HPV strains that were not specifically covered by the Cobas 4800 test. Remarkably, only 4 out of the 1052 samples (0.4%) showed no presence of HPV. In contrast, 91.6% of CIN3+ patients had previously tested positive for HPV using cytology. This underscores the high sensitivity of the standard HPV screening test within the context of the actual screening program, where it demonstrated an impressive sensitivity rate of 97.0%^17^. Author conducted a comprehensive study to assess the effectiveness of concurrent visual inspection with dilute acetic acid (VIA) or mobile colposcopy when compared to standalone high-risk human papillomavirus (hr-HPV) DNA testing (utilizing platforms like careHPV, GeneXpert, AmpFire, or MA-6000) in a real-world, resource-constrained setting. Additionally, author investigated the rate at which participants were subsequently lost to follow-up. The 'positivity' rates for EVA and VIA were 8.6% (95% CI, 6.7–10.6) and 2.1% (95% CI, 1.6–2.5), respectively, while the hr-HPV ‘positivity’ rate was 17.9% (95% CI, 16.7–19.0). It's noteworthy that a substantial majority of women in the cohort tested negative for both hr-HPV DNA and visual inspection (3588 out of 4482, or 80.1%). A smaller percentage, 2.1% (95% CI, 1.7–2.6), tested hr-HPV-negative but positive on visual inspection. In total, 51 women in the group tested positive on both measures. Out of the 274 individuals who tested positive for hr-HPV in a standalone test, a significant proportion, 191 (69.5%), returned for at least one follow-up visit^18^. Author study explores four distinct subsets: breast vs. cervical cancer, internal vs. external validation, comparing mammography, ultrasound, cytology, and colposcopy, and assessing the performance of deep learning (DL) algorithms versus human doctors. Based on a comprehensive analysis of 35 studies that met the inclusion criteria for this systematic review, author found that the pooled sensitivity stands at 88% (95% CI 85–90%), the specificity at 84% (79–87%), and the Area Under the Curve (AUC) at an impressive 0.92 (0.90–0.94)^19^.

To diagnose cervical cancer effectively, this study explores a wide array of both online and offline machine learning algorithms using benchmarked datasets. Additionally, hybrid techniques are employed to address segmentation challenges, and the feature count is optimized through the incorporation of tree classifiers. Remarkably, certain algorithms can attain accuracy, precision, recall, and F1 scores exceeding 100% as the training data percentage increases. While approaches such as logistic regression with L1 regularization can indeed achieve 100% accuracy, it's worth noting that they may come at a higher computational cost in terms of CPU time compared to other methods that can still deliver a commendable 99% accuracy with significantly lower computational demands^20^. The author used supervised machine learning to detect cervical cancer at an early stage. Author trained a machine learning model using a dataset from UCI that contains information related to cervical cancer. To assess how well our classifiers performed and how accurate they were, we trained them with and without a feature selection process. The author employed various feature selection methods, including Relief rank, the wrapper approach, and LASSO regression. Impressively, when we used all the features, the XG Boost model achieved a high accuracy rate of 94.94%. Interestingly, in some cases, the feature selection techniques performed even better^21^. Author proposed, deep learning plays a pivotal role in two distinct approaches. The first approach involves using pre-trained feature extractor models along with machine learning algorithms to classify cervical cancer images. In this case, ResNet-50 achieves the highest classification accuracy of 92.03%. The second approach employs transfer learning, where Google Net outperforms other models with a remarkable classification accuracy of 96.01%^22^. Author proposed a deep feature-fed MLP neural network and it incorporates four innovative ideas to adjust the number of neurons in its hidden layers. Additionally, the MLP is enhanced by inputting features extracted from ResNet-34, ResNet-50, and VGG-19 deep networks. The technique involves these two CNNs discarding their classification layers and passing their output through a flatten layer before inputting into the MLP. Both CNNs are trained with the Adam optimizer using relevant images to improve their performance. Remarkably, this proposed approach achieves outstanding results, with an accuracy of 99.23% for two classes and 97.65% for seven classes when evaluated on the Herlev benchmark database^23^. Table 1, illustrates the detailed comparison information of various state-of-the-methods. In the realm of cervical cancer detection and classification, there exists a noticeable research gap that centers around the need for enhanced methodologies. Current approaches often face limitations in terms of accuracy and robustness. To address this gap, our proposed research aims to leverage the power of deep learning models and their fusion to create a more comprehensive and effective system. By amalgamating the strengths of different deep learning architectures, our research seeks to improve the precision and reliability of cervical cancer detection, ultimately contributing to early diagnosis and better patient outcomes. This exploration into the fusion of deep learning models represents a novel avenue in the pursuit of advancing cervical cancer detection and classification techniques.

**Table 1:** Detailed information of state-of-the-art methods.

Author	Year	Dataset	Method	Outcome
^14^	2021	Intel and Smartphone ODT	VGG19, CYENET	92.4% of Se, 96.2% of Sp, and 88% of kappa
^15^	2023	SIPaKMeD	CNN	Obtained an accuracy of 91.13%
^16^	2022	Tercha General Hospital	MobileNetv2-YOLOv3	Acc of 96.84%
^17^	2021	NCBI	CNN	Acc of 97.04%
^18^	2023	CHB-ERC	CNN	Acc of 95.0%
^19^	2022	SIPaKMeD	Deep learning	Se of 88%, Sp of 84%, AUC of 0.92
^20^	2023	DTU/Herlev Pap smear	Stochastic average gradient	Acc of 99%
^21^	2023	UCI repository	Artificial neural network with XG Boost	Acc of 94.94%
^22^	2023	Pap smear	Google Net	Acc of 96.01%
^23^	2023	ImageNet	multi-layer perceptron	Acc of 97.65%

## Material and methods

### Dataset description

**Figure 2:**
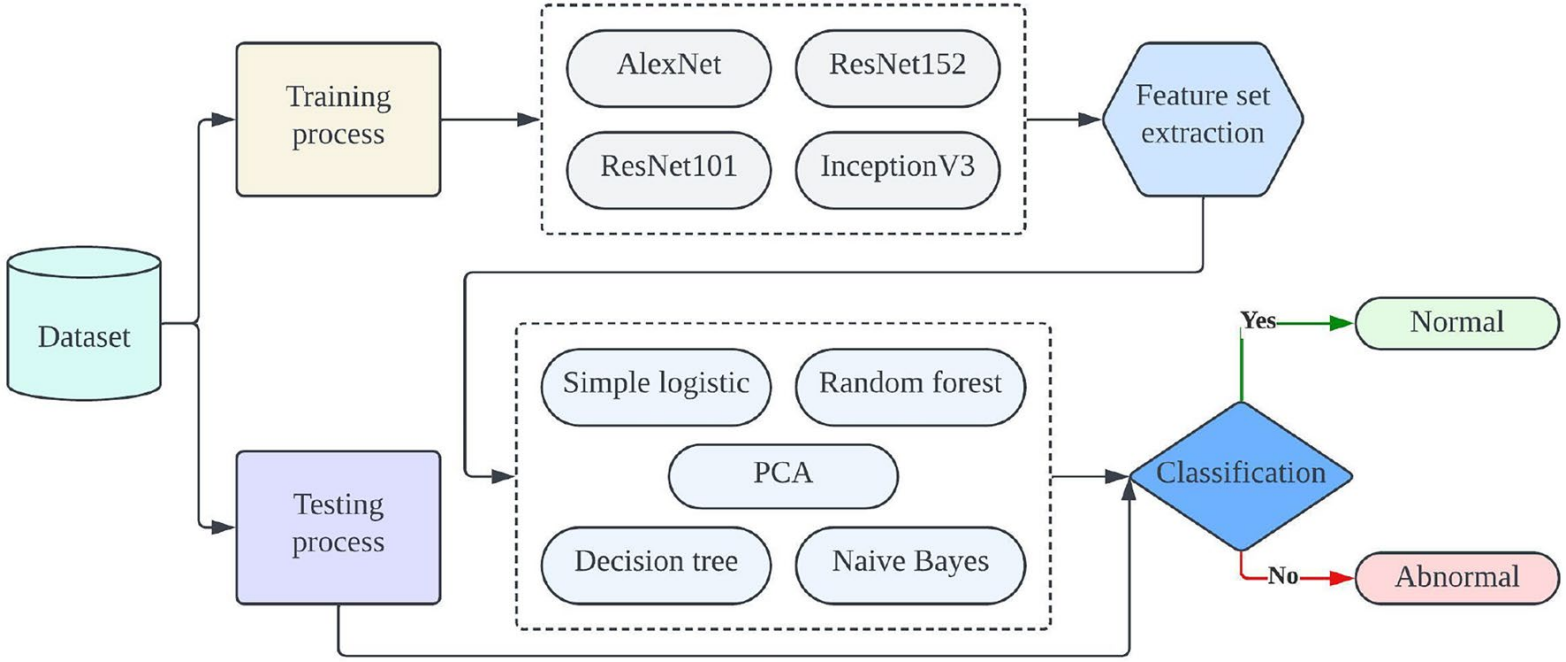
Proposed model cervical cancer classification.

The dataset we used for this study is accessible through this link: https://www.cs.uoi.gr/~marina/sipakmed.html. It contains five different cell types, as detailed in^24^. In our research, we've transformed this dataset into a two-class system with two categories: normal and abnormal. Specifically, the normal category includes superficial intermediate cells and parabasal cells, while the aberrant category covers koilocytotic, dyskeratotic, and metaplastic cell types^25^. Within the normal category, we've further divided cells into two subcategories: superficial intermediate cells and parabasal cells. The essential dataset characteristics are summarized in Table 2. The SIPaKMeD dataset comprises a total of 4068 images, with 3254 allocated for training (making up 80% of the total), and 813 set aside for testing (accounting for 20% of the total). This dataset consists of two distinct classes: normal photos, totalling 1618, and aberrant images, amounting to 2450. Figure 2 provides visual examples of photographs from these two different categories. The existing literature extensively covers different screening methods for cervical cancer, such as Pap smear, colposcopy, and HPV testing, emphasizing the importance of early detection. However, a significant gap exists in automated screening systems using pap smear images. Traditional methods rely on expert interpretation, but integrating deep learning (DL) and machine learning (ML) offers potential for intelligent automation. Despite this potential, few studies focus on developing and evaluating such systems specifically for cervical cancer prediction using pap smear images. This research addresses this gap by proposing a methodology that utilizes pre-trained deep neural network models for feature extraction and applies various ML algorithms for prediction. The study aims to contribute to advancing automated screening systems for cervical cancer, aiming to improve early detection and patient outcomes.

**Table 2:** SIPaKMeD dataset description.

Total images	Training	Testing	Normal image	Abnormal image
4068	3254	813	1618	2450

### Methods

The schematic representation of our proposed system can be observed in Fig. 2. To facilitate the classification task for cervical cancer, we employ the SIPaKMeD dataset, which comprises images of pap smears. This dataset is categorized into two groups: abnormal and normal, with a distribution of 60% for training and 40% for testing. To extract relevant feature sets from well-established CNN architectures such as Alexnet, Resnet-101, Resnet-152, and InceptionV3, we initiate feature extraction from these pretrained CNN models. This step allows us to gather valuable information from the final layer activation values. For the task of classifying images into normal and abnormal categories, we leverage a variety of machine learning techniques, including Simple Logistic, Decision Tree, Random Forest, Naive Bayes, and Principal Component Analysis. Our approach is designed as a hybrid strategy, merging both DL and ML methodologies. The utilization of DL enables our model to capture intricate and complex features inherent in the data, while ML provides the necessary flexibility to handle diverse scenarios. By harnessing the last layer of pretrained models for feature extraction, we enable different machine learning algorithms to classify data based on these extracted attributes. This combination of DL and ML enhances our system's ability to effectively categorize cervical cancer cases.

#### Pre-trained neural networks

The pre-trained model has undergone training on a larger dataset, acquiring specific weights and biases that encapsulate the dataset's distinctive characteristics. This model has been effectively employed for making predictions based on data. The transferability of learned features to other datasets is possible because certain fundamental abstract properties remain consistent across various types of images. By utilizing pre-trained models, significant time and effort savings are achieved, as a substantial portion of the feature extraction process has already been completed. Noteworthy examples of pre-trained models include Resnet152, ResNet101, Inceptionv3, and Alexnet, which are summarized in Table 3 for reference.

**Table 3:** Details of pre-trained models.

Year	Architecture	Input size	Layer size	No. of convolution	No. of parameters
2016	ResNet101	224 × 224	101	72	60 million
2016	ResNet152	224 × 224	152	106	60 million
2015	InceptionV3	229 × 229	48	48	25 million
2012	AlexNet	256 × 256	7	5	62.3 million

##### ResNet101

**Figure 3:**
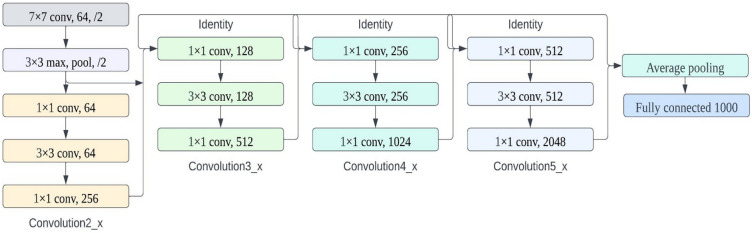
ResNet101 architecture.

The image classification framework based on ResNet-101 consists of two main parts: feature extraction and feature classification. In Fig. 3, you can see how the feature extractor is built, comprising five main convolution modules with a total of one hundred convolution layers, an average pooling layer, and a fully connected layer^26^. Once the features are extracted, they are used to train a classifier with a Softmax structure. Table 4 lists the convolution layers and their configurations in the ResNet-101 backbone. Using shortcut connections to increase data dimensions, the ResNet-101 model significantly improves performance by increasing convolutional depth. These shortcut connections also address the problem of network depth causing degradation by enabling identity mapping. For most binary classification tasks, the loss function is applied using the logical cross-entropy function, as shown in Eq. (1).

1 $$k_{({h_l},\;{q_l})}^b = - {f_l}\log \left( {q_l} \right) - \left( {1 - {f_l}} \right)\log \left( {1 - {q_l}} \right)$$

where the ground truth value, $\% {f_l}$, and the predicted value, $\% {q_l}$, are respectively indicated as the *l*th training dataset's ground truth and predicted values. The value of the loss, ${k}_{({h_{l}}, \; {q_{l}})}^{b}$, is then backpropagated through the CNN model. At the same time, the CNN model parameters (weights and biases) are gradually optimised during each epoch. This process continues until the loss is minimised and the CNN model converges to a solution.

**Table 4:** ResNet-101 configurations.

No. of layers	Size of output	Convolution2_x	Convolution3_x	Convolution4_x	Convolution5_x	Pooling layer
Size of output	112 × 112 × 64	56 × 56 × 56	28 × 28 × 128	14 × 14 × 256	7 × 7 × 512	1 × 1 × 512
No of filters	7 × 7, 64, /2	3 × 3, 64, /2	3 × 3, 128, /2	3 × 3, 256, /2	3 × 3, 512, /2	Avg

##### ResNet152

**Figure 4:**
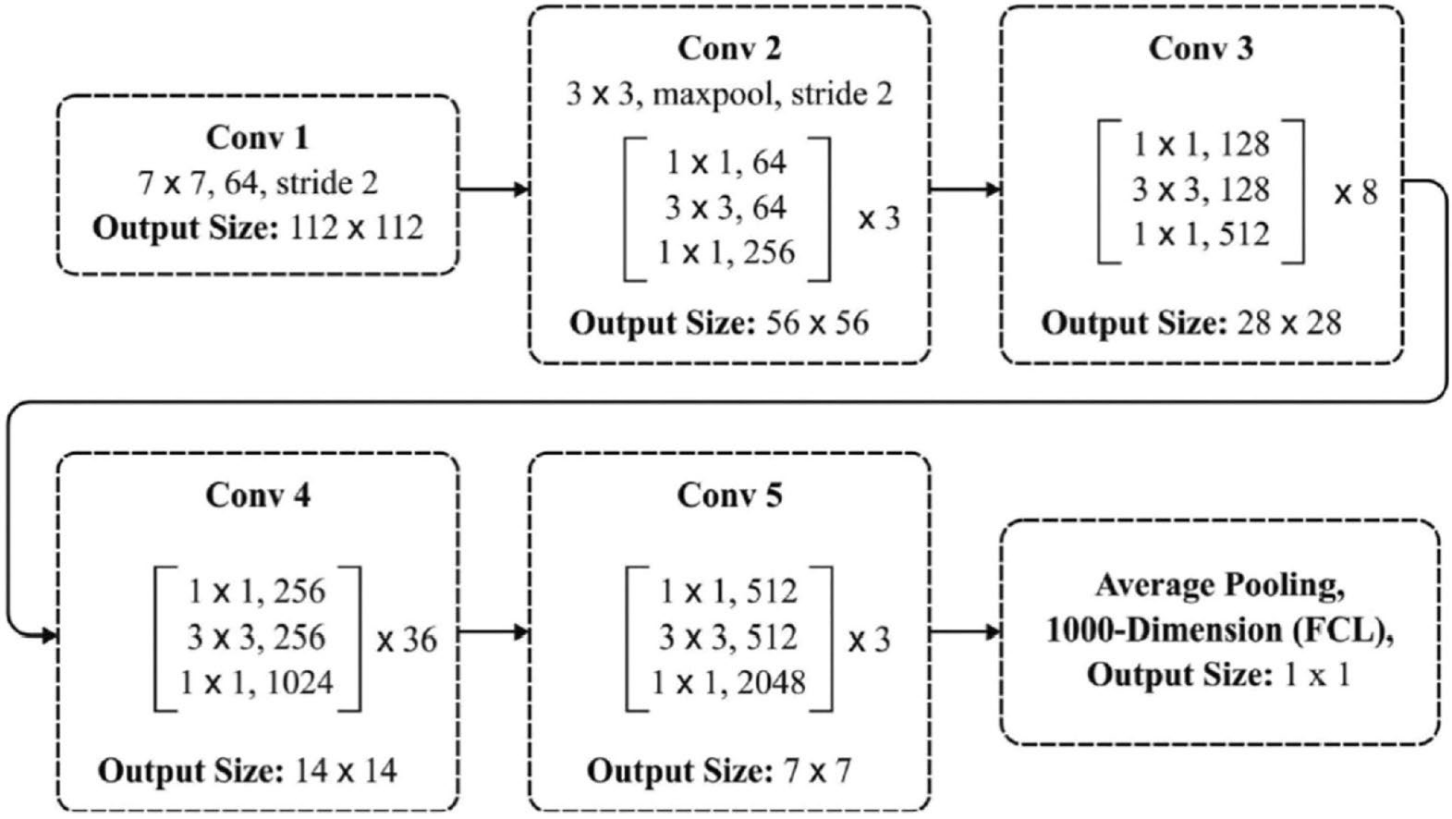
ResNet152 architecture.

The ResNet architecture is efficient, promoting the training of very deep neural networks (DNN) and enhancing accuracy. It addresses the challenge of accuracy degradation associated with increasing network depth. When depth is increased, accuracy often drops, which is a drawback. However, deeper networks can improve accuracy by avoiding the saturation of shallow networks, where errors remain minimal^27^. The key idea here is that information from one layer should easily flow to the next with the help of identity mapping. ResNet tackles the degradation problem, along with the gradient vanishing issue, using residual blocks. These blocks handle the remaining computation while considering the input and output of the block. Figure 4, illustrates architecture of ResNet152. Table 5, illustrates the configuration of ResNet152.

**Table 5:** ResNet-152 configurations.

No. of Layers	Size of output	Convolution2_x	Convolution3_x	Convolution4_x	Convolution5_x	Pooling layer
Size of output	112 × 112 × 64	56 × 56 × 128	28 × 28 × 256	14 × 14 × 512	7 × 7 × 1024	1 × 1 × 2048
No of filters	7 × 7, 64, /2	3 × 3, 128, /2	3 × 3, 256, /2	3 × 3, 512, /2	3 × 3, 1024, /2	Avg

##### InceptionV3

**Figure 5:**
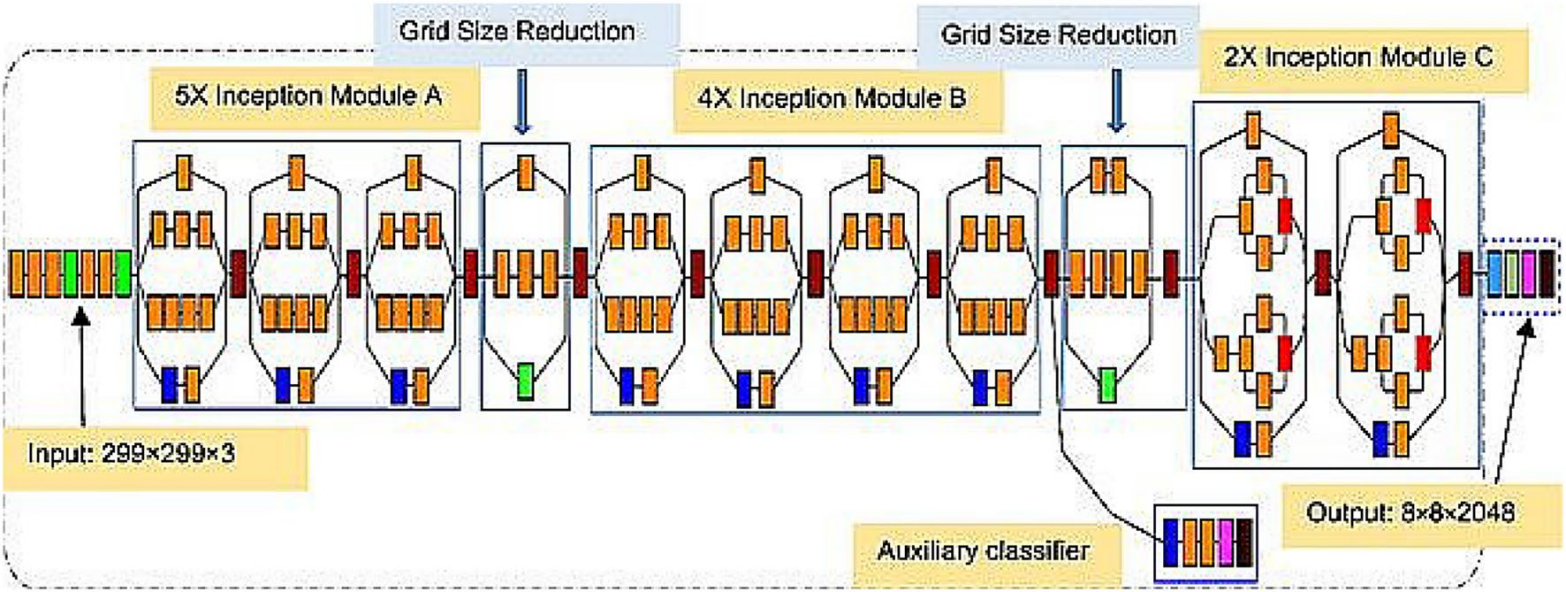
InceptionV3 architecture.

This advanced model has undergone training by one of the industry's most renowned hardware specialists, leveraging an impressive repertoire of over 20 million distinct parameters. The model's architecture is a harmonious blend of symmetrical and asymmetrical construction blocks, each meticulously crafted with its own unique set of convolutional, average, and maximum pooling layers, concatenation operations, and fully connected layers configurations. Furthermore, the model's design incorporates an activation layer that takes advantage of batch normalization, a widely adopted technique in the field. This technique helps stabilize and accelerate the training process, making the model more robust and efficient^28^. For the critical task of classification, the model employs the Softmax method, a popular and well-established approach in machine learning. Softmax is instrumental in producing probability distributions over multiple classes, which enables the model to make informed and precise predictions. To provide a visual understanding of the Inception-V3 model's intricate design, Fig. 5 serves as a diagrammatic representation, offering insights into the model's underlying architecture and the various components that make it a powerhouse in the realm of machine learning and artificial intelligence.

##### AlexNet

The field of machine learning, particularly in the domain of image processing, has witnessed a profound impact thanks to the advent of Alexnet. As suggested in Ref.^29^, this influential model boasts a preconfigured Convolutional Neural Network (CNN) with a total of eight distinct layers^29^. Its remarkable performance in the 2012 ImageNet Large Scale Visual Recognition Challenge (LSVRC-2012) competition marked a watershed moment, as it clinched victory with a substantial lead over its competitors. The architectural blueprint of Alexnet bears some resemblance to Yann Lecun's pioneering LeNet, highlighting its historical lineage and the evolutionary progress of convolutional neural networks.

**Figure 6:**
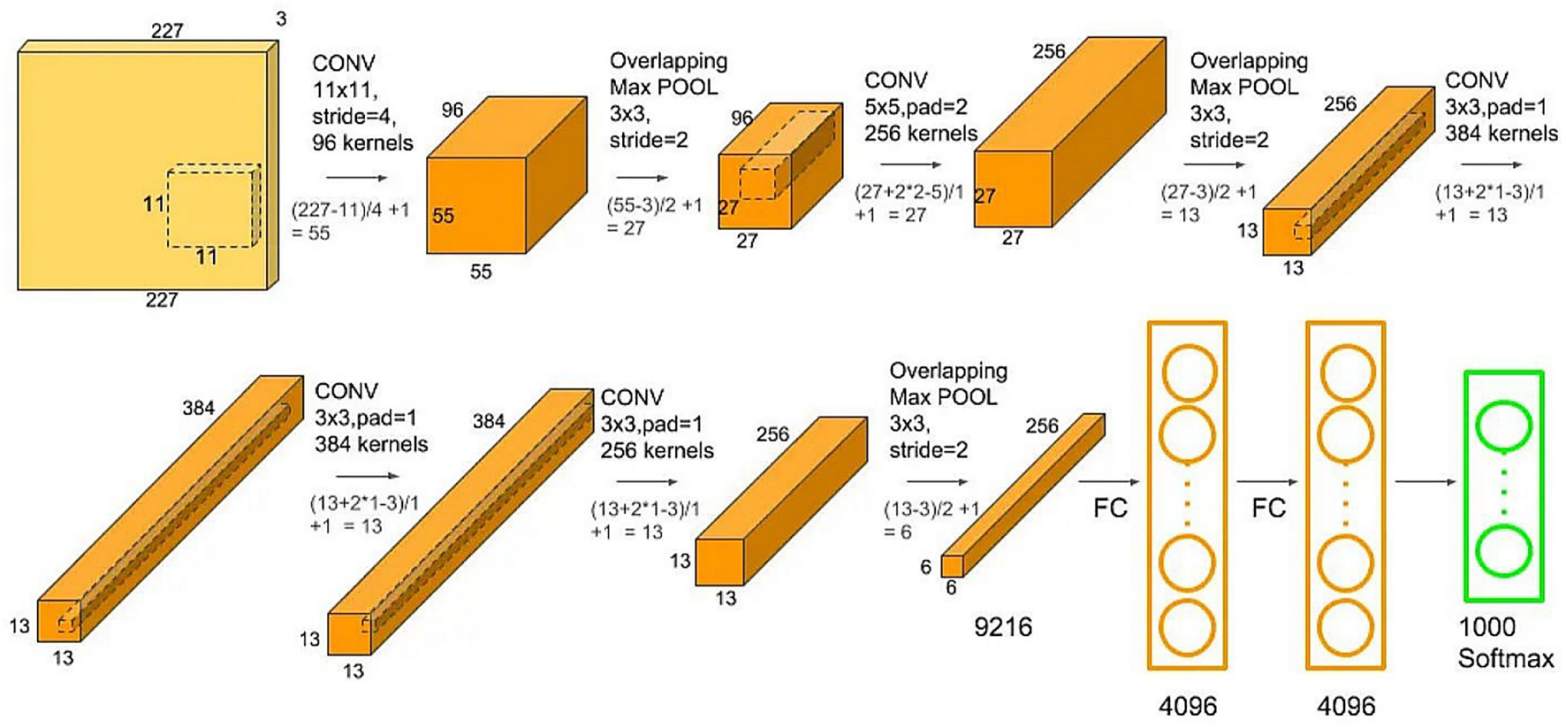
AlexNet architecture.

Figure 6 provides an insightful visual representation of the holistic design of the Alexnet system. In the journey of data processing within Alexnet, input data traverse through an intricate sequence, comprising five convolution layers and three max-pooling layers, as vividly illustrated in Fig. 5. These layers play a pivotal role in feature extraction and hierarchical representation, which are vital aspects of image analysis and understanding. The culmination of AlexNet's network journey is marked by the application of the SoftMax activation function in the final layer, enabling it to produce probabilistic class predictions. Along the way, the Rectified Linear Unit (ReLU) activation function is systematically employed across all the network's convolution layers, providing a nonlinear transformation that enhances the network's capacity to learn and extract features effectively. This combination of architectural elements and activation functions has played a significant role in solidifying AlexNet's position as a groundbreaking model in the domain of image processing and machine learning.

##### Simple logistic regression

Logistic regression serves as a powerful method for modelling the probability of a discrete outcome based on input variables, making the choice of input variables a pivotal aspect of this modelling process. The most common application of logistic regression involves modelling a binary outcome, which pertains to scenarios where the result can exclusively assume one of two possible values, such as true or false, yes or no, and the like. However, in situations where there are more than two discrete potential outcomes, multinomial logistic regression proves invaluable in capturing the complexity of the scenario. Logistic regression finds its primary utility in the realm of classification problems^30^. It becomes particularly valuable when the task at hand involves determining which category a new sample best aligns with. This becomes especially pertinent when dealing with substantial datasets, where the need to classify or categorize data efficiently and accurately is paramount. One noteworthy domain where logistic regression finds widespread application is in cybersecurity, where classification challenges are ubiquitous. A pertinent example is the detection of cyberattacks. Here, logistic regression plays a crucial role in identifying and categorizing potential threats, contributing significantly to bolstering the security of digital systems and networks.

##### Decision tree

In the realm of supervised learning algorithms, decision trees emerge as a highly versatile and powerful tool for both classification and regression tasks. They operate by constructing a tree-like structure, wherein internal nodes serve as decision points, branches represent the outcomes of attribute tests, and terminal nodes store class labels. The construction of a decision tree is an iterative process, continually dividing the training data into subsets based on attribute values until certain stopping conditions, such as reaching the maximum tree depth or the minimum sample size required for further division, are met. To guide this division process, the Decision Tree algorithm relies on metrics like entropy or Gini impurity, which gauge the level of impurity or unpredictability within the data subsets^31^. These metrics inform the algorithm’s choice of the most suitable attribute for data splitting during training, aiming to maximize information gain or minimize impurity. In essence, the central nodes of a decision tree represent the features, the branches encapsulate the decision rules, and the leaf nodes encapsulate the algorithm’s outcomes. This design accommodates both classification and regression challenges, making decision trees a flexible tool in supervised machine learning. One notable advantage of decision trees is their effectiveness in handling a wide range of problems. Moreover, their ability to be leveraged in ensembles, such as the Random Forest algorithm, enables the simultaneous training on multiple subsets of data, elevating their efficacy and robustness in real-world applications.

##### Random forest

A Random Forest is a powerful machine learning tool that handles both regression and classification tasks effectively. It works by combining the predictions of multiple decision trees to solve complex problems. Here's how it works: The Random Forest algorithm builds a “forest” of decision trees using a technique called bagging. Bagging improves the precision and reliability of machine learning ensembles^32^. The algorithm then makes predictions by averaging the results from these trees, determining the final outcome. What makes the Random Forest special is its scalability. Unlike single decision trees, it can adapt to complex data and improves its accuracy as you add more trees to the “forest.” The Random Forest also helps prevent overfitting, making it a valuable tool for real-world applications with noisy and complex datasets. Moreover, it reduces the need for extensive fine-tuning, making it an appealing choice for practitioners seeking effective and dependable machine learning models.

##### Naïve Bayes

Naïve Bayes theorem forms the fundamental principle underlying the Naive Bayes algorithm. In this method, a key assumption is that there's no interdependence among the feature pairs, resulting in two pivotal presumptions: feature independence and attribute equality. Naive Bayes classifiers are versatile, existing in three primary variants: Gaussian Naive Bayes, Bernoulli Naive Bayes, and Multinomial Naive Bayes^33^. The choice of variant depends on the nature of the data being analyzed. For binary data, Bernoulli Naïve Bayes is employed, while count data finds its match in Multinomial Naïve Bayes, and continuous data is aptly handled by Gaussian Naïve Bayes. Equation (2) serves as a proof of Bayes theorem, underpinning the mathematical foundations of this approach.

2 $$Z\left( {b|a} \right) = \frac{Z\left( b \right)Z\left( b \right)}{{Z\left( a \right)}}$$

##### Principal component analysis

Principal Component Analysis (PCA) serves as a powerful technique designed to mitigate the impact of correlations among variables through an orthogonal transformation. PCA finds widespread use in both exploratory data analysis and machine learning for predictive modelling. In addition, PCA stands out as an unsupervised learning algorithm that offers a valuable approach for delving into the intricate relationships between variables. This method, also referred to as generic factor analysis, enables the discovery of the optimal line of fit through regression analysis^34^. What sets PCA apart is its ability to reduce the dimensionality of a dataset without prior knowledge of the target variables while preserving the most critical patterns and interdependencies among the variables. By doing so, PCA simplifies complex data, making it more amenable for various tasks, such as regression and classification. The result is a more streamlined subset of variables that encapsulates the essential essence of the data.

## Experimental results and discussion

Feature extraction in this process relies on pre-trained models. Some notable examples of these models include ResNet101, ResNet152, InceptionV3, and AlexNet. For classification purposes, machine learning techniques like Simple Logistic, Decision Tree, Random Forest, Naive Bayes, and Principal Component Analysis (PCA) come into play. Among these models, the ResNet152 feature extraction method stands out for its exceptional performance, achieving the highest testing accuracy at 99.08%. When it comes to machine learning, the Simple Logistic model outperforms all the pre-trained models in terms of accuracy. It's worth noting that there's a slight but noticeable gap between training accuracy and testing accuracy for all the pre-trained models, with training accuracy consistently higher. This discrepancy underscores the complexities of classifying cells associated with cervical cancer.

**Figure 7:**
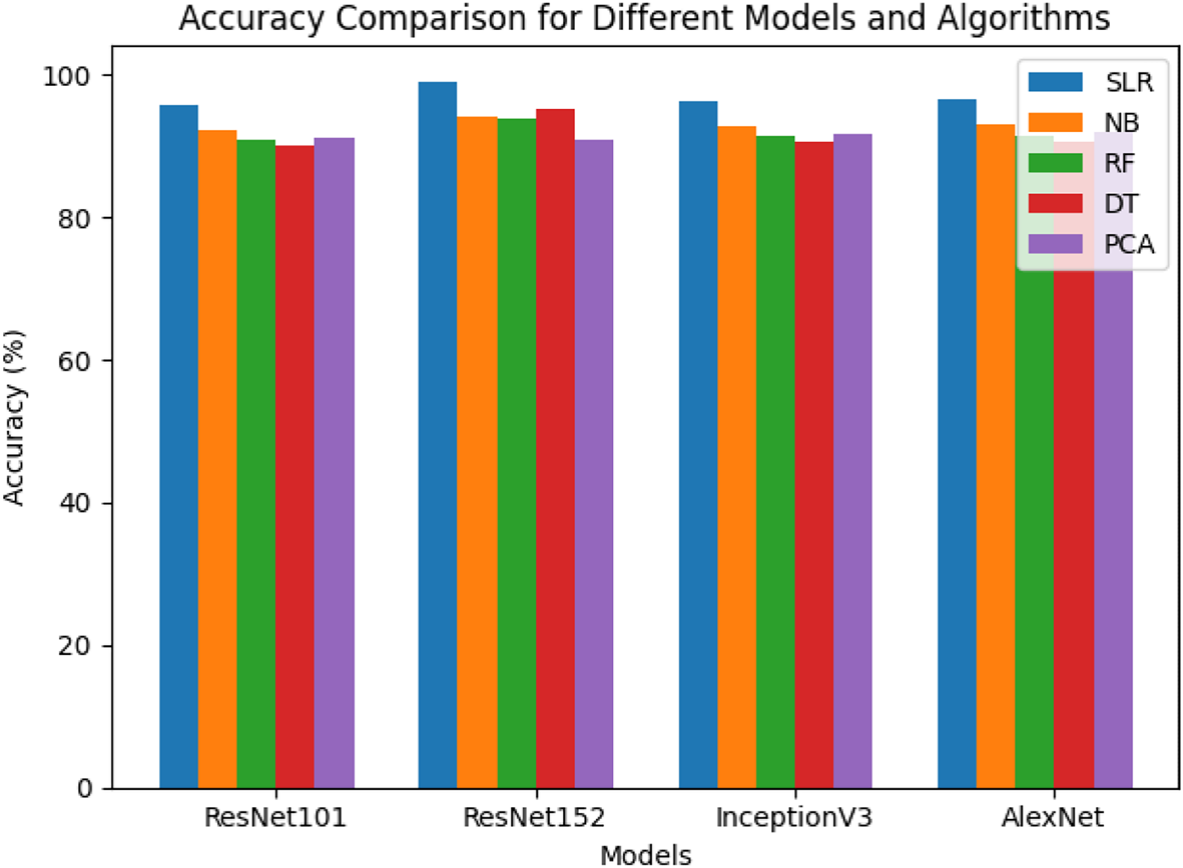
Pretrained models accuracy (%) comparison.

Leveraging a variety of machine learning approaches proves significant, as it not only provides flexibility but also improves accuracy in tackling this challenging classification task. The subsequent sections will delve into the findings obtained using the suggested method, offering a comprehensive comparison of the employed pre-trained models in Table 6. While ResNet152 achieved the highest accuracy, it's essential to highlight that the Simple Logistic Classifier, with the highest accuracy among all the pre-trained models, will be the focal point of this discussion. Additionally, Fig. 7 provides a visual comparative analysis of all the pre-trained models, revealing the performance of the Simple Logistic Classifier as the benchmark for reference.

**Table 6:** Classification accuracy comparison of pre-trained models.

No. of classifiers	ResNet101	ResNet152	InceptionV3	AlexNet
SLR	95.81	98.08	95.01	96.31
NB	90.09	94.29	89.29	90.59
RF	91.73	95.93	90.93	92.23
DT	86.65	90.85	85.85	87.15
PCA	92.02	95.22	91.22	92.52

The ResNet-101 model plays a pivotal role in feature extraction. It's worth noting that a range of machine learning techniques, including Simple Logistic, Decision Tree, Random Forest, Naive Bayes, and Principal Component Analysis, were applied to both the test and training datasets. For a comprehensive view of the results, Table 7 present the outcomes for the training and testing sets, respectively. When compared to other classifiers, the Random Forest stood out, achieving the highest accuracy (Acc) (98.62%), precision (Pr) (98.64%), recall (98.64%), and the lowest mean absolute error (0.062%) for the training dataset. On the other hand, the test dataset yielded the best results with simple logistic regression, which delivered an accuracy of 95.81%, precision of 94.41%, recall of 94.32%, and a root mean square of 0.225.

**Table 7:** Pre-trained ResNet101 training and testing phases. Significant values are in bold.

No. of classifiers	ResNet101—Training phase					ResNet101—Testing phase				
	Precision (%)	Recall (%)	MAE	RMSE	Accuracy (%)	Precision (%)	Recall (%)	MAE	RMSE	Accuracy (%)
SLR	95.81	95.92	0.1	0.206	95.74	94.41	94.32	0.113	0.225	**95.81**
NB	90.22	89.33	0.122	0.342	89.32	89.92	88.97	0.125	0.358	90.09
RF	98.64	98.62	0.062	0.14	**98.62**	90.54	90.53	0.198	0.298	91.73
DT	89.52	89.57	0.232	0.322	89.49	85.55	85.54	0.259	0.359	86.65
PCA	90.01	89.74	0.07	0.162	97.39	90.82	90.81	0.125	0.309	92.02

The ResNet-152 model helps extract features. Various machine learning techniques, like Simple Logistic, Decision Tree, Random Forest, Naive Bayes, and Principal Component Analysis, were used on both the test and training data. Table 8 show the results for the training and testing sets. Among the classifiers, Random Forest performed the best, with the highest accuracy (98.98%), precision (99.64%), recall (99.42%), and the lowest mean absolute error (0.053%) for the training data. In contrast, for the test data, simple logistic regression delivered the best results with an accuracy of 98.08%, precision of 95.41%, recall of 94.21%, and a root mean square of 0.22.

**Table 8:** Pre-trained ResNet152 training and testing phases. Significant values are in bold.

No. of classifiers	ResNet152—Training phase					ResNet152—Testing phase				
	Precision (%)	Recall (%)	MAE	RMSE	Accuracy (%)	Precision (%)	Recall (%)	MAE	RMSE	Accuracy (%)
SLR	96.61	96.72	0.091	0.202	98.81	95.21	95.12	0.11	0.22	**98.08**
NB	91.02	90.13	0.113	0.338	90.13	90.72	89.77	0.122	0.353	94.29
RF	99.44	99.42	0.053	0.136	**98.98**	91.34	91.33	0.195	0.293	95.93
DT	90.32	90.37	0.223	0.318	90.3	86.35	86.34	0.256	0.354	90.85
PCA	90.81	90.54	0.061	0.158	98.2	91.62	91.61	0.122	0.304	95.22

The Inceptionv3 model handles feature extraction, and we used several machine learning techniques like Simple Logistic, Decision Tree, Random Forest, Naive Bayes, and Principal Component Analysis on both the training and test datasets. Table 9 show the results for both training and testing. Among these methods, the Random Forest performed the best for training, with the highest accuracy (97.74%), precision (97.75%), recall (97.73%), and the lowest mean absolute error (0.068%). On the other hand, for the test data, simple logistic regression delivered outstanding results with an accuracy of 98.08%, precision of 95.01%, recall of 93.42%, and a root mean square of 0.2349.

**Table 9:** Pre-trained InceptionV3 training and testing phases. Significant values are in bold.

No. of classifiers	InceptionV3—Training phase					InceptionV3—Testing phase				
	Precision (%)	Recall (%)	MAE	RMSE	Accuracy (%)	Precision (%)	Recall (%)	MAE	RMSE	Accuracy (%)
SLR	94.92	95.03	0.106	0.294	94.86	93.51	93.42	0.117	0.2349	**95.01**
NB	89.33	88.44	0.128	0.43	88.44	89.02	88.07	0.129	0.3679	89.29
RF	97.75	97.73	0.068	0.228	**97.74**	89.64	89.63	0.202	0.3079	90.93
DT	88.63	88.68	0.238	0.41	88.61	84.65	84.64	0.263	0.3689	85.85
PCA	89.12	88.85	0.076	0.25	96.51	89.92	89.91	0.129	0.3189	91.22

**Figure 8:**
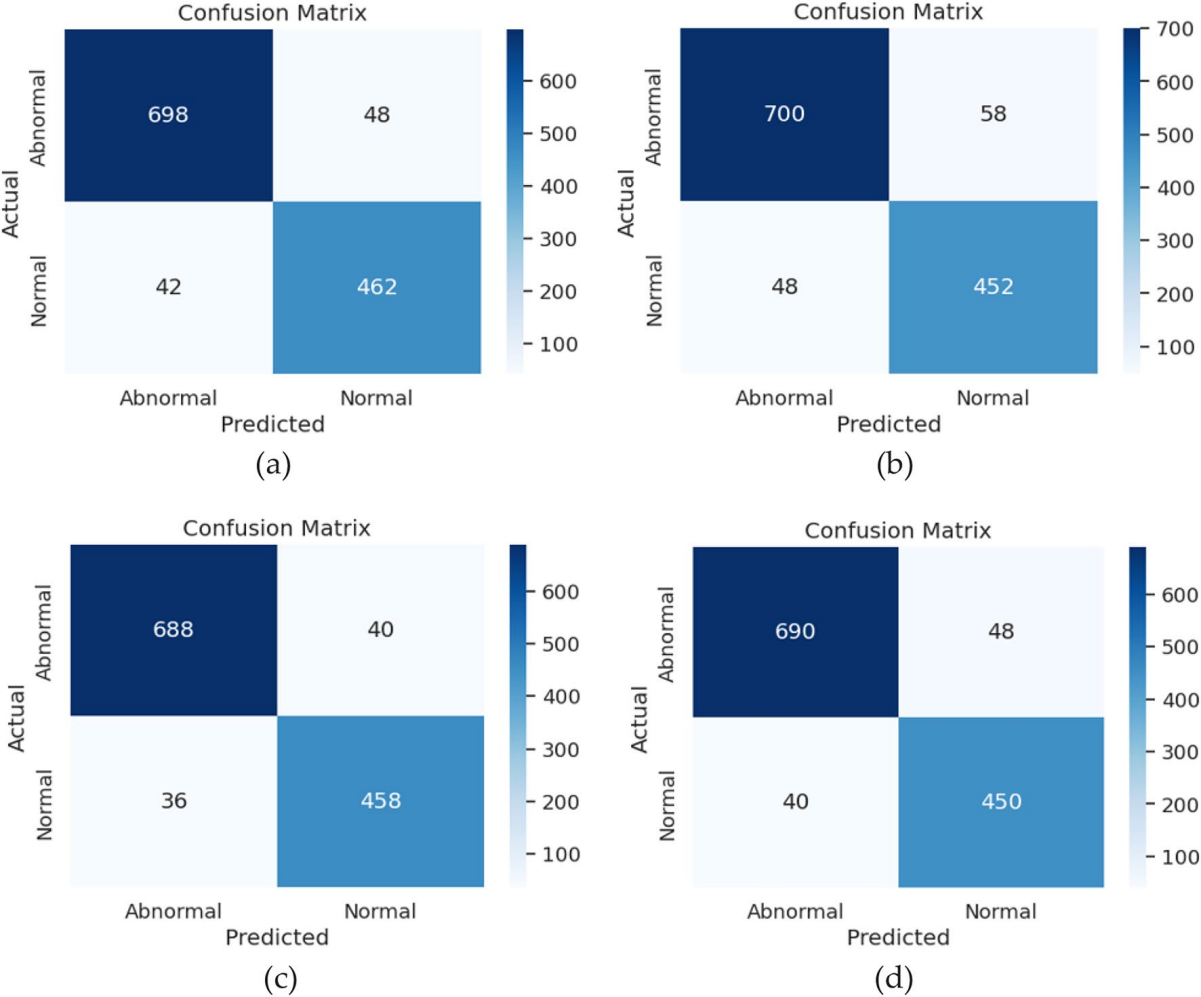
Confusion matrix for (**a**) ResNet101; (**b**) ResNet152; (**c**) InceptionV3; (**d**) AlexNet.

The task of feature extraction is managed by the AlexNet model, and we employed a range of machine learning techniques, such as Simple Logistic, Decision Tree, Random Forest, Naive Bayes, and Principal Component Analysis, on both the training and test datasets. Table 10 present the findings for both training and testing phases. Among these methodologies, the Random Forest excelled during training, achieving the highest levels of accuracy (99.12%), precision (98.83%), recall (98.81%), and the lowest mean absolute error (0.061%). Conversely, for the test data, simple logistic regression yielded remarkable results, attaining an accuracy of 96.31%, precision of 94.81%, recall of 94.72%, and a root mean square of 0.234. Figure 8, illustrates the confusion matrix of all four pre-trained models.

**Table 10:** Pre-trained AlexNet training and testing phases.

No. of classifiers	AlexNet—Training phase					AlexNet—Testing phase				
	Precision (%)	Recall (%)	MAE	RMSE	Accuracy (%)	Precision (%)	Recall (%)	MAE	RMSE	Accuracy (%)
SLR	96	96.11	0.0998	0.204	96.24	94.81	94.72	0.1128	0.234	96.31
NB	90.41	89.52	0.1218	0.34	89.82	90.32	89.37	0.1248	0.357	90.59
RF	98.83	98.81	0.0618	0.138	99.12	90.94	90.93	0.1978	0.297	92.23
DT	89.71	89.76	0.2318	0.32	89.99	85.95	85.94	0.2588	0.358	87.15
PCA	90.2	89.93	0.0698	0.16	97.89	91.22	91.21	0.1248	0.308	92.52

**Figure 9:**
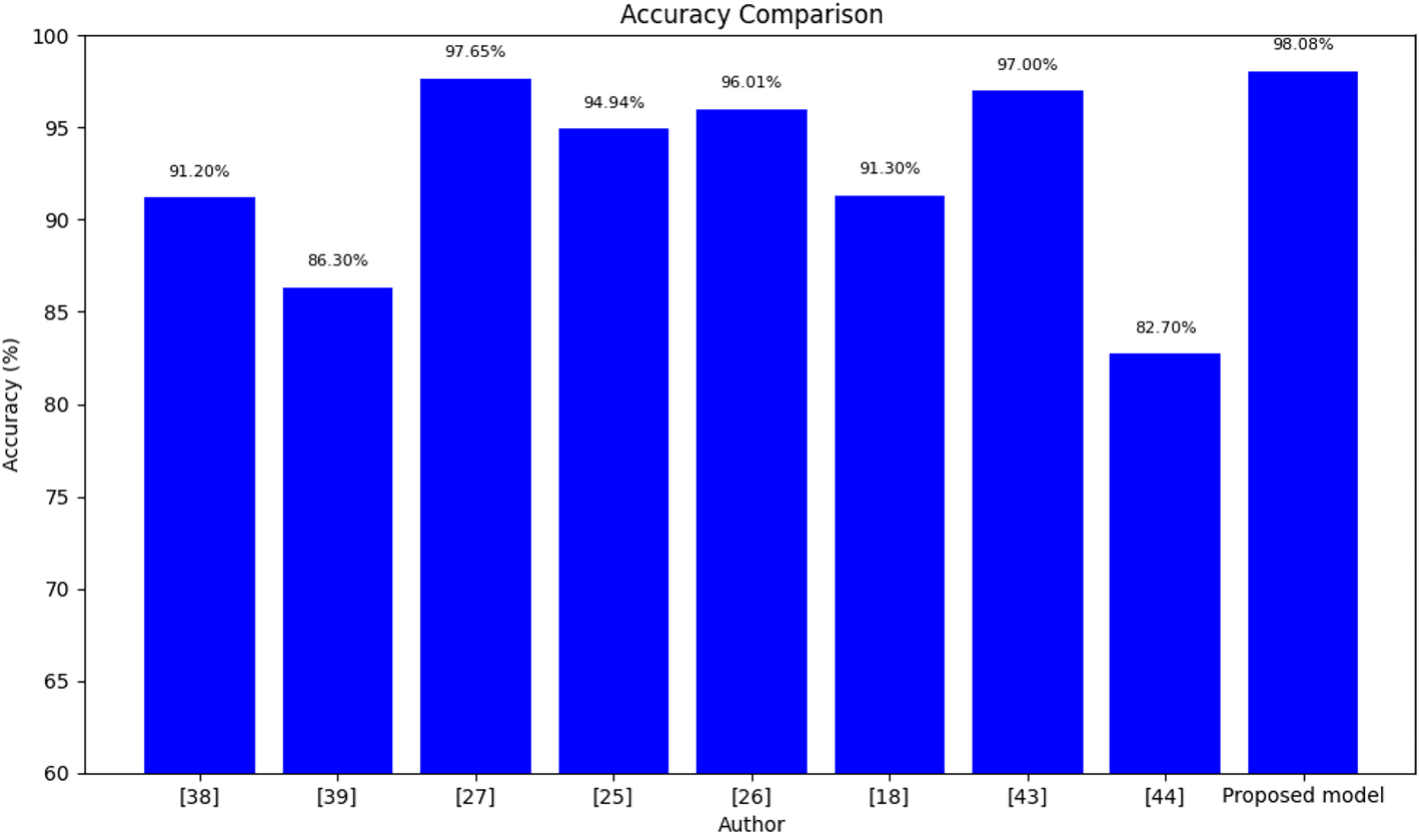
Graphical illustration of accuracy comparison of proposed and other models.

Table 11 illustrates a comprehensive comparison of classification accuracy between the proposed ResNet152 model with SLR and several state-of-the-art models. The table presents a detailed breakdown of the performance metrics, emphasizing the accuracy of these models in solving the specific task. The accuracy values are expressed as percentages, showcasing how effectively each model can correctly classify data points. The proposed ResNet152 with SLR stands out by achieving the highest classification accuracy of 98.08%. This remarkable result demonstrates the effectiveness of this model in comparison to other state-of-the-art models. Figure 9 complements the insights from Table 11 by offering a visual representation of the accuracy comparison. This bar chart clearly illustrates how the proposed ResNet152 with SLR outperforms other existing models in terms of accuracy. Each bar in the chart represents a different model, with its height corresponding to its accuracy percentage. The striking difference in the height of the bar for the proposed model highlights its superior performance, with an accuracy of 98.08%. This figure serves as a powerful visualization of the comparison, making it easy for readers to grasp the extent of the proposed model's excellence. Together, Table 11 and Fig. 9 provide a robust and easily interpretable analysis of the model comparison, emphasizing the outstanding performance of the proposed ResNet152 with SLR in the context of accuracy. These visual aids are essential in conveying the significance of the research findings to your readers and stakeholders.

**Table 11:** Classification accuracy comparison of proposed and state-of-the-art models.

Author	Model	Accuracy (%)
Manal Darwish et al.^35^	Enhanced with shifted patch tokenization	91.20
Peng et al.^36^	VGG16	86.30
Shervan Fekri-Ershad et al.^24^	MLP + CNN	97.65
Gaurav Kumawat^22^	ANN + 6 classifiers	94.94
Madhura et al.^23^	ResNet50	96.01
Shtwai Alsubai et al.^15^	Hybrid CNN	91.30
Bryar Shareef et al.^37^	ESTAN	97.0
Bryar Shareef et al.^38^	Hybrid-MT-ESTAN	82.7
Proposed model	Pre-trained CNN models	98.08

## Conclusion

Cervical cancer classification is a challenging task within the medical field. Interpreting and diagnosing cervical cancer have historically been a complex process for pathologists. However, advancements in technology have led to a shift in how this task is approached. Specifically, the integration of Deep Learning (DL) and Machine Learning (ML) algorithms has emerged as a powerful tool, enhancing the precision of cervical cancer classification. Central to this progress is the use of pretrained models such as ResNet101, ResNet152, InceptionV3, and AlexNet. These models have been carefully fine-tuned through training on pap smear images, allowing them to effectively extract key features from the intricate world of Pap smear images. Our innovative approach, applied to the SIPaKMeD dataset, represents a pioneering method that combines the strengths of DL and ML in the field of cervical cancer classification. DL excels at extracting intricate features from images, providing a foundation for various ML algorithms to work with. This hybrid methodology holds promise for improving cervical cancer classification. The results obtained from our approach are indeed promising. For instance, ResNet101 achieved an accuracy of 95.81%. However, ResNet152 stands out as the leading model, achieving an impressive accuracy of 98.08%. Notably, these exceptional results were achieved using Simple Logistic classifiers, which outperformed other classification techniques. Furthermore, when Simple Logistic is combined with pretrained DL models, it reaffirms its effectiveness as the top-performing ML approach, highlighting the strength and efficiency of this combination in the context of cervical cancer classification. This not only confirms the potential of this model but also underscores the promising prospects of our hybrid DL-ML approach for advancing the field of cervical cancer diagnosis. In conclusion, these findings emphasize the transformative potential of our hybrid methodology. By skilfully combining DL and ML, we aim to contribute to the ongoing evolution of cervical cancer diagnosis, ultimately improving patient outcomes. Our results point toward a future where cervical cancer classification is more accurate, efficient, and accessible.

## Data Availability

The datasets used during the current study https://www.cs.uoi.gr/~marina/sipakmed.html.
